# Single-subject assessment of the distribution of white matter abnormalities measured by diffusion tensor imaging in patients with severe traumatic brain injury

**DOI:** 10.1186/cc13659

**Published:** 2014-03-17

**Authors:** S Magnoni, M Macri, F Stretti, C Mac Donald, R Biffi, E Zanier, A Snyder, J Shimony, N Stocchetti, D Brody

**Affiliations:** 1 Fondazione IRCCS Ca Granda - Ospedale Maggiore Policlinico, Milan, Italy; 2 Milan University, Milan, Italy; 3 Washington University, St Louis, MO, USA; 4 IRCCS - Istituto di Ricerche Farmacologiche Mario Negri, Milan, Italy

## Introduction

Traumatic axonal injury (TAI) is a major contributor to adverse outcomes following traumatic brain injury (TBI). Diffusion tensor imaging (DTI) is a magnetic resonance imaging technique, which provides a robust measure of white matter integrity in patients with TBI. Certain brain regions have been identified as particularly susceptible to TAI by means of DTI. Few studies, however, have focused on describing the distribution of DTI abnormalities in individual TBI patients. The aim of this project was to conduct an exploratory analysis of the extent and distribution of axonal injury in TBI patients at a singlesubject level.

## Methods

Patients admitted to the ICU for severe TBI underwent brain MRI and DTI (32 directions, *b *= 1,000, voxel size 2 x 2 x 2 mm^3^) between 2 weeks and 3 years after injury. For each individual patient, we enrolled five age-and-sex-matched healthy volunteers who underwent the same DTI protocol and were used as controls (31 total). We used a region of interest (ROI) automated analysis [1] to quantify white matter integrity. The fractional anisotropy (FA) maps were segmented using a white matter parcellation map covering the entire brain. Our primary outcome was the normalized difference in FA between each patient and the controls. Abnormalities were defined as values that were more than 2 SD below the mean of the values for the matched controls for each ROI.

## Results

Twelve TBI patients with a median age of 30 years (range 20 to 38) and a median GCS of 5 (range 5 to 7) were included. The majority of them had diffuse axonal injury according to CT and conventional MRI. Analysis of individual ROIs revealed that all subjects had numerous abnormal ROIs, varying from 14 to 64 (median 21) out of 78 regions assessed. The most frequently altered regions were the frontal lobe, orbital-frontal regions, corpus callosum, internal capsules, superior and inferior longitudinal fasciculi, cingulum, cerebellar structures and corona radiata. Thalamus, hippocampus and occipital lobes were less frequently affected.

## Conclusion

The extent and distribution of TAI varies on a patient basis. These findings highlight the need for standardized methods to precisely assess TAI in single subjects. Such single-subject methods will probably improve prognostic accuracy and may be useful in clinical trials.
